# Date pits based nanomaterials for thermal insulation applications—Towards energy efficient buildings in Qatar

**DOI:** 10.1371/journal.pone.0247608

**Published:** 2021-03-26

**Authors:** Moza Ghorab Al Marri, Mohammad A. Al-Ghouti, Vasanth C. Shunmugasamy, Nabil Zouari

**Affiliations:** 1 Department of Biological and Environmental Sciences, College of Arts and Sciences, Qatar University, Doha, State of Qatar; 2 Mechanical Engineering Program, Texas A & M University, Doha, Qatar; Mirpur University of Science and Technology, PAKISTAN

## Abstract

Air-conditioning systems make the most significant part of energy consumption in the residential sector. There is no denying that it is essential to produce a comfortable indoor thermal environment for residents in a building. The actual goal is to achieve thermal comfort level without putting too much cost on the ecological system by trying to conserve the amount of energy consumed. An effective way to help achieve such a goal is by incorporating thermal insulation in buildings. Thermal insulations help reduce thermal energy gained during the implementation of a desired thermal comfort level. This study aims to use an environmentally friendly nanoparticle of date pits to create thermal insulations that can be used in buildings. Different ratios of the nanoparticle of the date pits and sand composite were investigated. Fourier transform infrared spectroscopy and scanning electron microscopy were used to characterize the new materials. The material with nanoparticles of date pits and 50% by-volume epoxy provided good thermal insulation with thermal conductivity of 0.26 W⁄mK that could be used in the existing buildings. This has the potential to reduce the overall energy consumption by 4,494 kWh and thereby reduce CO_2_ emissions of a 570 m^2^ house by 1.8 tons annually. In conclusion, the future of using nanoparticles of date pits in construction is bright and promising due to their promising results.

## 1. Introduction

Qatar is considered as a developing country, with one of the fastest-growing economies in the world. It is involved in several infrastructure development megaprojects, including the hosting of the 2022 FIFA World Cup. Therefore, Qatar is continually facing a rising energy demand. In recent years, many countries, including Qatar have increasingly become aware of the risks posed by climate change. This has put much emphasis on reducing the use of fossil fuels, reducing energy consumption, and finding ways to use energy more efficiently [1]. Qatar is expected to consume up to 80,648 GWh of energy by 2020 and the energy demand will keep increasing [2]. It has been reported that the energy consumed in the Gulf Cooperation Council (GCC) countries is increasing significantly in the past 30 years [3].

Air-conditioning systems make the most significant part of energy consumption in the residential sector. It is estimated that about 80% of the energy in Qatari buildings is utilized to provide thermal comfort to the occupants. This number is extremely high; in fact, this is the most significant fraction in comparison to other countries. Providing a suitable indoor thermal environment is crucial, however, it should be achieved by conserving the amount of consumed energy. Since thermal insulations help reduce thermal energy gained during the implementation of a desired thermal comfort level. Hence, incorporating thermal insulation in buildings is considered as a suitable method for the reduction of energy demand. A recent finding studied and analyzed the impact of three energy-efficient measures to assess their benefits on Qatari buildings [4]. Those measures included the addition of thermal insulation walls and roofs, the use of efficient lamps, and increasing the houses’ cooling temperature from 22 °C to 24 °C. The results indeed demonstrated a 46% decrease in the total cooling load of buildings. This implies that optimizing the use of energy can act as an appropriate contributor to energy-saving technologies, such as the case of thermal insulators [5,6]. The efficiency of a heat spreading material is related to its thermal conductivity, and Watts per meter-Kelvin (W/mK) is the normally used unit of thermal conductivity. Therefore, to attain an effective indoor thermal comfort condition, the application of suitable materials would be required for efficient building envelope design.

The generation of waste, greenhouse emissions, and global energy consumption should be taken into consideration to focus on energy efficiency and control of emissions in the building sector. Today, the demand for sustainable "green" practices is imposing significant pressure on industries regarding their utilization of synthetic or natural materials and their energy usage for building and conservation [7]. The production of very fine particle sizes ranging from 1–100 nm is being used to improve the material and the design of the system on a smaller scale [7,8]. Using insulation in buildings for the upcoming years will be the main focal point regarding the demand for efficient buildings. The problem with heat loss and heat gain of buildings is the fact that the materials used such as glass surfaces and low material insulators are posing an issue of energy conservation [9].

Thermal insulation with the application of nanomaterials would be the best option for better thermal insulation quality in comparison to other traditional materials that can be applied [10]. There are several thermal insulations such as nansulate and aerogel [11]. Nansulate is used as a coating insulator technology that can form a nanocomposite known as HydroNM-Oxide. This nanotechnology offers low thermal conductivity; therefore, it is an outstanding insulator. The other insulator has characteristics that make it highly suitable for insulation. Aerogel is known to have a relatively low density with nanoporous materials that can be used with silica to form a nanocomposite with low thermal conductivity [11]. Examples of other nanomaterials that can be used for construction purposes are carbon nanotubes, titanium dioxide nanoparticles, silicon dioxide nanoparticles, and many others. The use of these nanomaterials as a composite with other materials, like cement, can result in a significant decrease in carbon dioxide emissions and the performance of thermal insulations will be enhanced to obtain an efficient use of energy [8,12–15].

**Fig 1:**
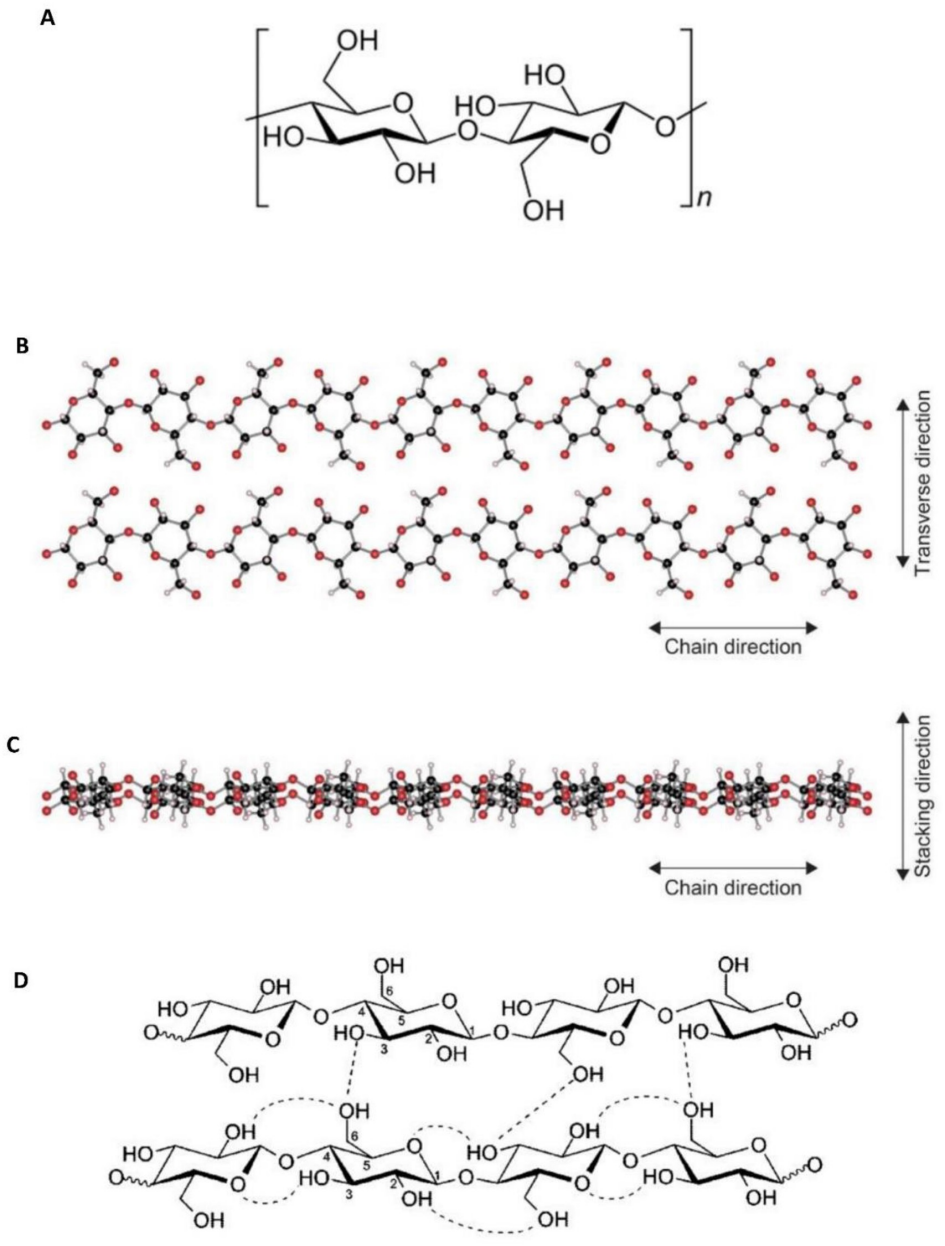
A. Cellulose structure, B and C. Cellulose structure observed along two directions [18], and D. Intra- and intermolecular hydrogen bonds in cellulose [20].

Cellulose, the most abundant polysaccharide in nature, is bio-based materials that are photosynthesized and accumulated in plants. Because of the availability of date pits and biodegradability, it has been generating much activity in the materials science field [16]. Date pits are considered suitable materials due to their macrostructure, physical and chemical properties such as insolubility in water, high mechanical strength, chemical stability, and economic viability [17]. Fig 1A shows that the cellulose structure is a linear chain of the ringed glucose molecule, arranged in a crystalline and amorphous like structure. Recently, composites containing cellulose have gained attention as potential heat spreading materials and could be appropriate in thermal management [18]. Table 1 details some of the most prominent physical and chemical characteristics of the date pit [19].

**Table 1:** Physical and chemical characteristics of date pits [19].

Characteristic	Values
Calcium content	0.31%
Oxygen content	7.93%
Sulfur content	0.45%
Others total metals	1.39%
Carbon content (% on dry basis)	84.01%
Cellulose	41.7%
Lignin	16.9%
Hemicellulose	10.2%
Total surface area	99.76 m^2^/g
Pore diameter	33.3 nm
Cumulative pore volume	0.140 cm^3^/g

Thermal insulation is one of the applications of using cellulose [21]. Cellulose composed of the flat ribbon-like chains are stacked in parallel (Fig 1B and 1C) [18]. The network of hydrogen bonds and the inter-sheet stability stabilize the cellulose sheets. This could be due to out-of-plane van der Waals interactions between the backbone ring structures Fig 1D [20]. The weak bonding results in a lower thermal conductivity. In general, general building insulators are characterized by a value of less than 0.1 W/mK thermal conductivities [21]. However, the high-performance aerogels have lower thermal conductivities of 0.0262 W/mK at 300 K under 0.1 MPa. A study demonstrated the use of natural materials, such as date palm wood, in the production of thermal insulations for buildings [5]. It has been concluded that the relative permittivity was considerably affected by the fiber orientation of the date palm wood. As a result, date palm wood was an appropriate sample to be used in an efficient and safe insulating material. Another study used date pit powder with polystyrene to form a DPP-polystyrene composite that exhibits an effective thermal insulator material with properties that are similar to the conventional insulating materials that are typically used [22].

Therefore, this study aims to prepare a low-cost and environment-friendly material from natural by-products such as date pits, as an effective thermal insulator material for buildings. Date pits are characterized by macrostructure, chemical stability, water insolubility, high mechanical strength, and economic viability. Around 755 thousand tons of date pits as wastes are generated worldwide [23,24].

## 2. Materials and methods

### 2.1 Materials collection and preparation

A representative sample of date pits was collected from the local market. To remove impurities, the sample was rinsed several times with deionized water. After that the sample was dried for 24 hrs at 100 °C, then it was roasted at 130°C for 5 hrs. The roasted date pits (DP) were crushed and grounded into powder using a grinder to obtain particle size ranging from coarse particles to fine particles. The particle size ranged from 0.250 mm to 0.125 mm, then it was further processed to nanoscale using the MSK-SFM-1 Bench-Top Planetary Automatic Ball Mills at 50 Hz for 3 hrs [25]. The obtained new materials were referred to as nanoparticles of DP (nano-DP) in this study. The nanosize of the sample was checked using scanning electron microscopy (SEM) and transmission electron microscopy (TEM).

The sand sample (S) was collected from the dune’s surface from southeastern Qatar at 25.0856°N and 51.3689°E. The sand particles were then processed to nanoscale using the same ball mill at 50*Hz* for 3 hrs. The nanosize of the sand sample was also checked using SEM and TEM. Different mixing ratios were prepared between nano-DP and S to form DP-S mixtures, namely DP0S, DP10S, DP20S, DP30S, and DP40S with the following weight (wt%) ratios 100:0, 90:10, 80:20, 70:30, 60:40, respectively. The final weight of each DP-S mixture was 20 g.

Then, the prepared mixtures (DP0S, DP10S, DP20S, DP30S, and DP40S) were continuously mixed with a ratio of 1:1 of epoxy (E)/hardener, for 3 minutes to achieve uniform mixtures. The hardener-to-epoxy ratio was optimally 1:7, which is required to achieve adequate strength. The final mixture was poured into a cylindrical test mold, which has a radius of 12.25 mm and a height of 21 mm and left to air cure in the lab with an average temperature of 22.5 ± 0.5°C and 45 ± 5% relative humidity. The following composites were named as DP0S50E, DP10S50E, DP20S50E, DP30S50E, and DP40S50E. For instance, DP10S50E means that the weight (wt%) ratio between the DP, S, epoxy (E) is 40:10:50. The final weight of each preparation was 30 g.

**Fig 2:**
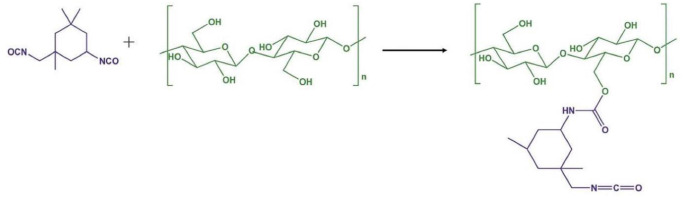
The reaction of the DP and the epoxy (E)/hardener [26].

In Fig 2, the isocyanate group (–NCO) was covalently bonded with hydroxyls (–OH) on the surface of the DP. To validate the DP and the epoxy (E)/hardener, Fourier transform infrared spectroscopy (FTIR) analysis was carried out.

### 2.2. Physical and chemical characterizations of the prepared materials

#### 2.2.1. Morphology, particle size analysis, and particle density

The morphology of the prepared materials was examined using the SEM (JEOL JSM-7600F), Energy Dispersive X-ray Spectroscopy (EDX), and TEM to determine its composition after mixing with epoxy [27]. The particle size analysis was performed using laser diffraction. The particle density of the samples was measured by pressing it into a known cylindrical mold’s dimension to reach a volume (Eq 1), the mold was tared to determine the sample’s weight using Eq 2, and the density was then determined using the Eq 3,

$$V=\pi r^{2}h$$ (1)

$$m_{\text{composition}}=m_{\left(\text{composition}+\text{mold}\right)}-m_{\text{mold}}$$ (2)

$$\rho =\frac{m}{v}$$ (3)

Where ρ is the density (kg⁄m^3^), m is the mass (kg), and V is the volume (m^3^).

#### 2.2.2. Fourier transform infrared spectroscopy (FTIR)

FTIR was performed to identify the different functional groups that are present on the material’s surface. A range of 400–4000 cm^-1^ was used in the scanning with a step size of 4 cm^-1^ and a scanning rate of 40 [28,29]. To prepare the measurement, the Hot Disk TPS 2500 S device was used. The sample was distributed on a Styrofoam plate. The plate’s depth, height, and width were 2.5 × 4 × 4 mm, respectively. During the measurement, the sensor was sandwiched inside the sample. The implemented output power to the sensor was 0.4 W.

#### 2.2.3. Optical microscopy

Light optical microscopy is a method in which the quantitative information of a material is captured [30]. Images of the specimen’s surface are captured by a calibrated reflected white LED light source at 50× total magnification with a digital pixel resolution using a Nikon Eclipse Model L200N. The photomicrographs of the composition of the samples were scanned using the Nikon Eclipse Model L200N to determine the DP compositions after mixing with epoxy.

#### 2.2.4. Thermal conductivity measurement

The test was carried out using a standard hot disc technique applied to the Hot Disk TPS 2500 S device that has a sensor that is fixed between two halves of specimens, and the experimental specification is set to similar previous values. The heat produced by the disc runs through the two halves of the sample, and the temperature of the sensor and samples were increasing over time. The rate by which the temperature increases depends on the sample’s materials; if the thermal conductivity of the sample is low, then the flow of temperature is high [31,32]. Fourier’s law Eq (4) describes the heat conduction (Q) across a slab of solid material.

$$Q=kA\frac{\Delta T}{Y}$$ (4)

**Fig 3:**
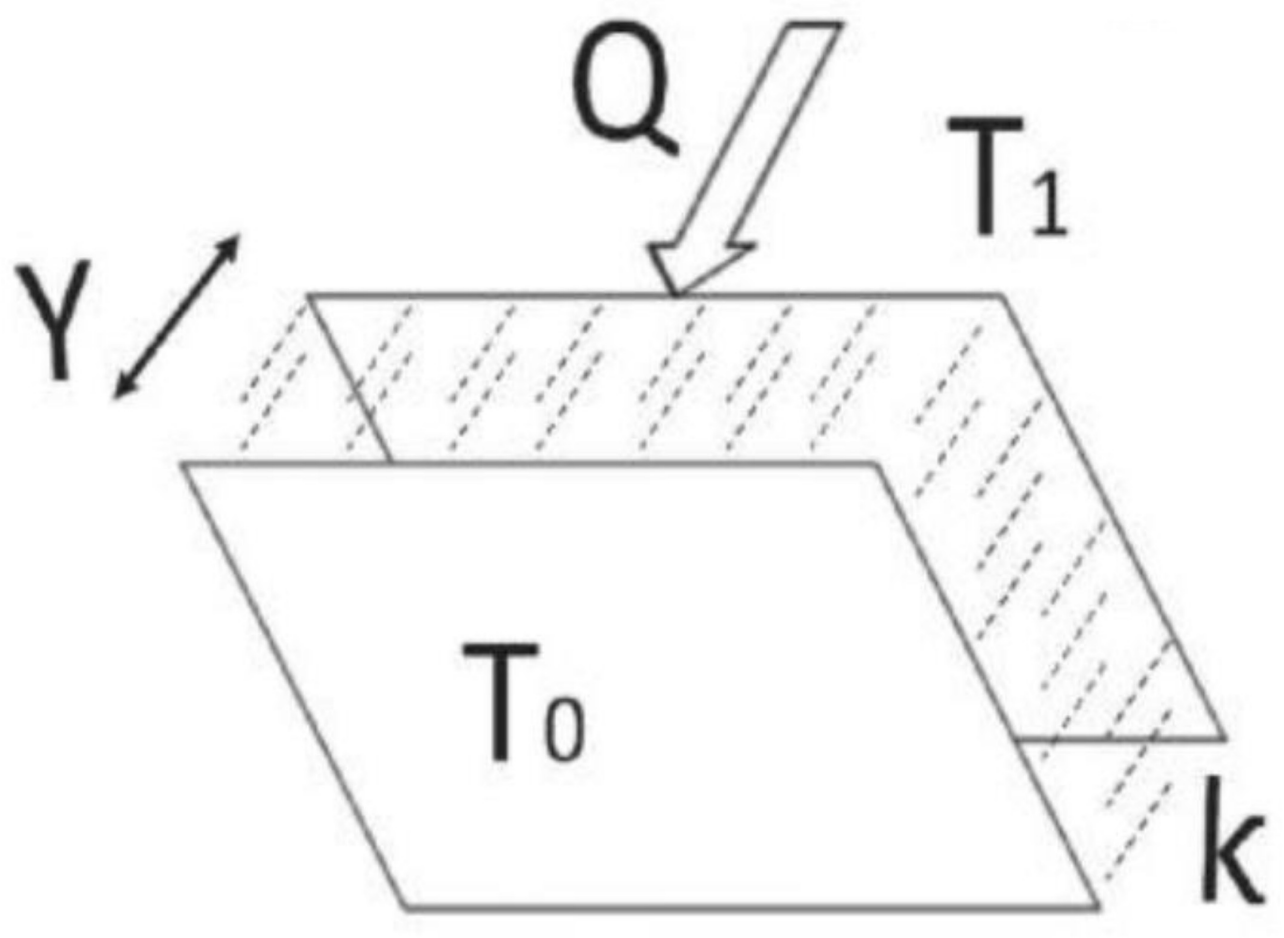
Heat conduction across a slab of a solid material [33].

Where, A is the cross-section surface area, Y is the thickness, k is the thermal conductivity of the material (in W/mK), ΔT is the different temperatures T1 and T0 between the two sides, respectively, as shown in Fig 3. For a steady-state condition, T remains constant, while for the homogeneous substances (non-equilibrium state), the T changes with time. In the case of a one-dimensional steady temperature field, the Q (heat flux) per unit time is proportional to the temperature gradient ΔT/Δx in the x-direction, and it is the thermal conductivity *k* is the proportional constant [21].

#### 2.2.5. Thermogravimetric analysis (TGA)

The thermogravimetric scales are used to characterize biomass; they evaluate the weight loss of a sample when temperature increases in a controlled environment. This environment could be an oxidizing atmosphere (e.g., air) or an inert atmosphere (e.g., nitrogen or helium) [34,35]. This method also allows investigators to observe the thermal stability of the specimen where the TGA is obtained. The T50 decomposition temperature is when the material loses 50% of its original mass [35]. The TGA analysis was done using the TGA 8000 in an inert atmosphere to measure the thermal stability of the biomass and the pyrolysis reaction. The TGA analyzer was raised from 0°C to 900°C at a rate of 10°C/min under a flow of nitrogen gas at 60 cm^3^⁄min.

#### 2.2.6. Ultraviolet (UV) aging test

UV exposure is a test performed in the laboratory to determine changes in the functional properties of a certain material and to define its aging mechanism [36]. Epoxy resin might deteriorate after long exposure to the environment, which would affect the reliability and the properties of the specimens [37]. A test involving long exposure of the material in-situ would take too much time, but accelerated aging methodologies can be applied to predict the durability of the material in a very short time [38,39]. In the accelerated aging test, the tested material was examined at a high aging rate by accelerating one of the affecting circumstances of the environment, and then the aging test parameters are transformed into real-time properties using mathematical equations. It is well known that the longevity of epoxy resin mainly depends on moisture diffusion, which can be obtained by the TGA test. In the case of coatings, this involves exposure to UV irradiation, which can be easily examined by using accelerated the aging test using QUV Accelerated Weathering Tester [40]. The laboratory UV accelerated aging test allows researchers to study the long-term effects and changes of material properties within a very short period of time as compared to actual outdoor weathering aging. Exposure levels were chosen to meet Qatari weather circumstances and to accelerate the aging process within the available testing time. As is well known, the most critical climate conditions that act as aging agents for building and construction materials are solar radiation and extreme temperatures [41]. The chosen sample that was examined by the aging test is DP0S50E. The specimens were cut into three long rectangular prisms of 10 cm, with a width of 5 mm. The QUV chamber was set to 60 °C with a maximum irradiation of UVA-340 (1.55 W⁄m^2^ @ 340 nm) and the specimens were exposed continuously for 2 days to simulate local harsh, extreme weather conditions.

### 2.3. Building thermal simulation

**Fig 4:**
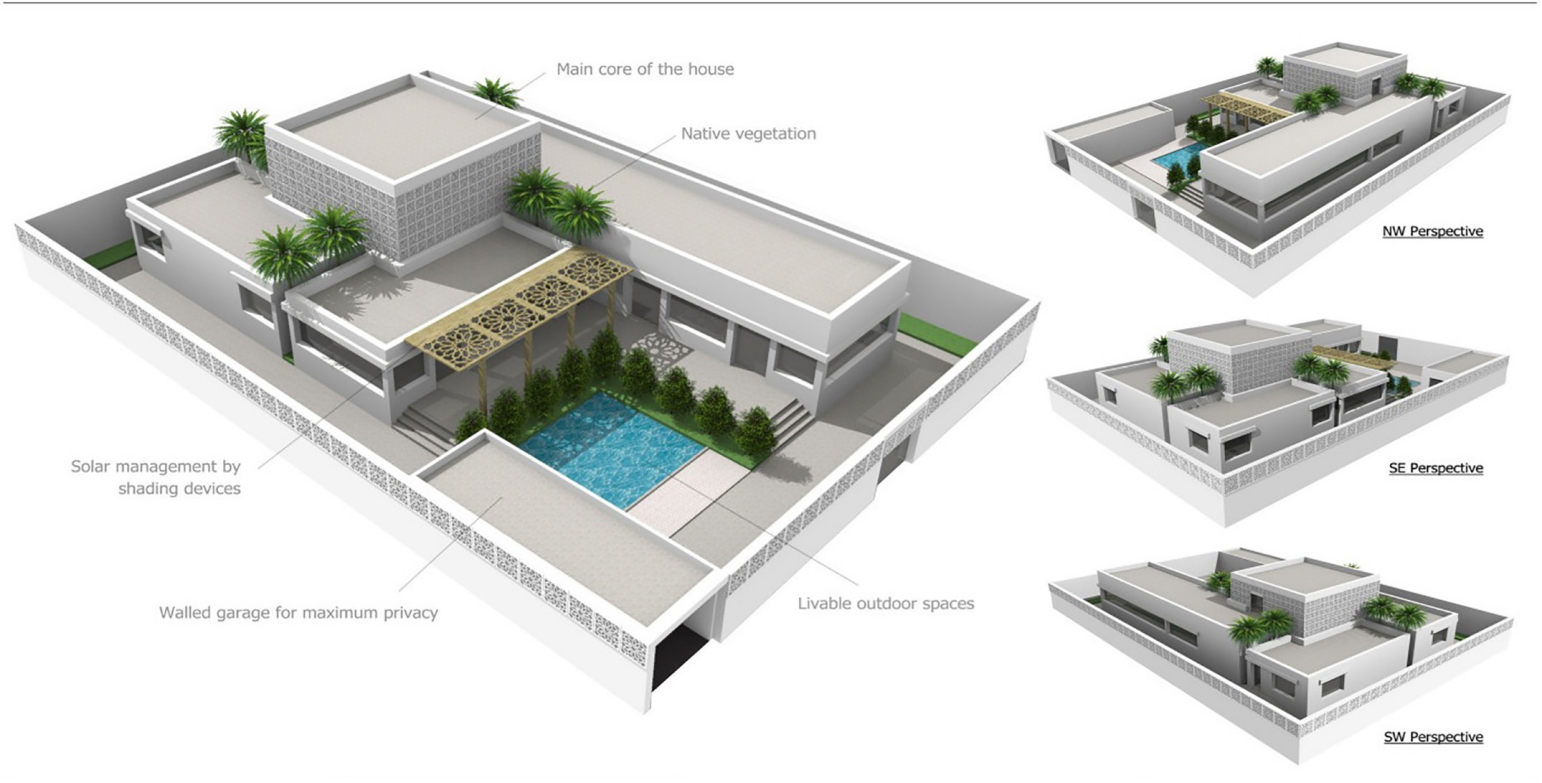
3D rendering of the conceptual house [42].

**Fig 5:**
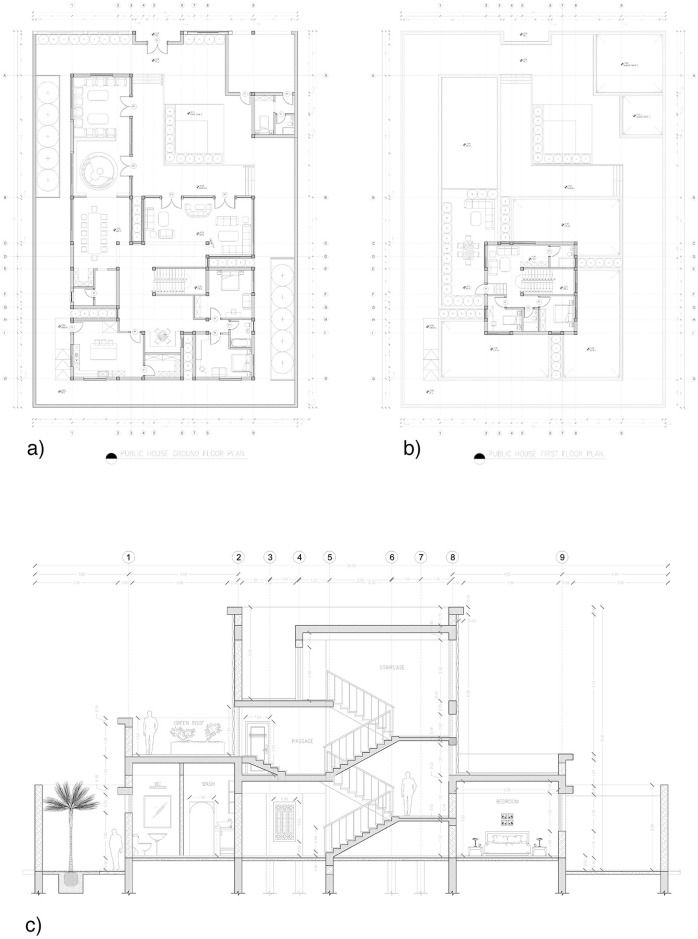
Architectural drawing of the house’s a) Ground Floor Plan, b) First Floor Plan, and c) Section Plan (all plans are not to scale) [42].

A conceptual residential home design was chosen to run the building energy simulation. The house was divided into five units: Majlis, living unit, main core, bedrooms, and services [42]. The area of the chosen house design is 570 m^2^. Some conceptual 3D renders by the architect are shown in Fig 4. The architectural plans of the house are given in Fig 5, showing the ground floor, first floor, and sectional plan of the house. The house design was then transferred to Revit software, which was associated with the building information modeling (BIM) system software. The model was capable of determining and visualizing the building’s energy performance with or without the proposed material of DP0S50E. A user interface plug-in is used in the Revit software to integrate the data to calculate the thermal properties of different building materials and structures, such as concrete walls, reinforced columns, reinforced beams, bitumen insulation paints, and plaster. The house’s location is chosen in the Baaya neighborhood in Ar-Rayyan.

## 3. Results and discussion

### 3.1. Physical and chemical characterizations

#### 3.1.1. Morphology of nanoparticle of DP particles

**Fig 6:**
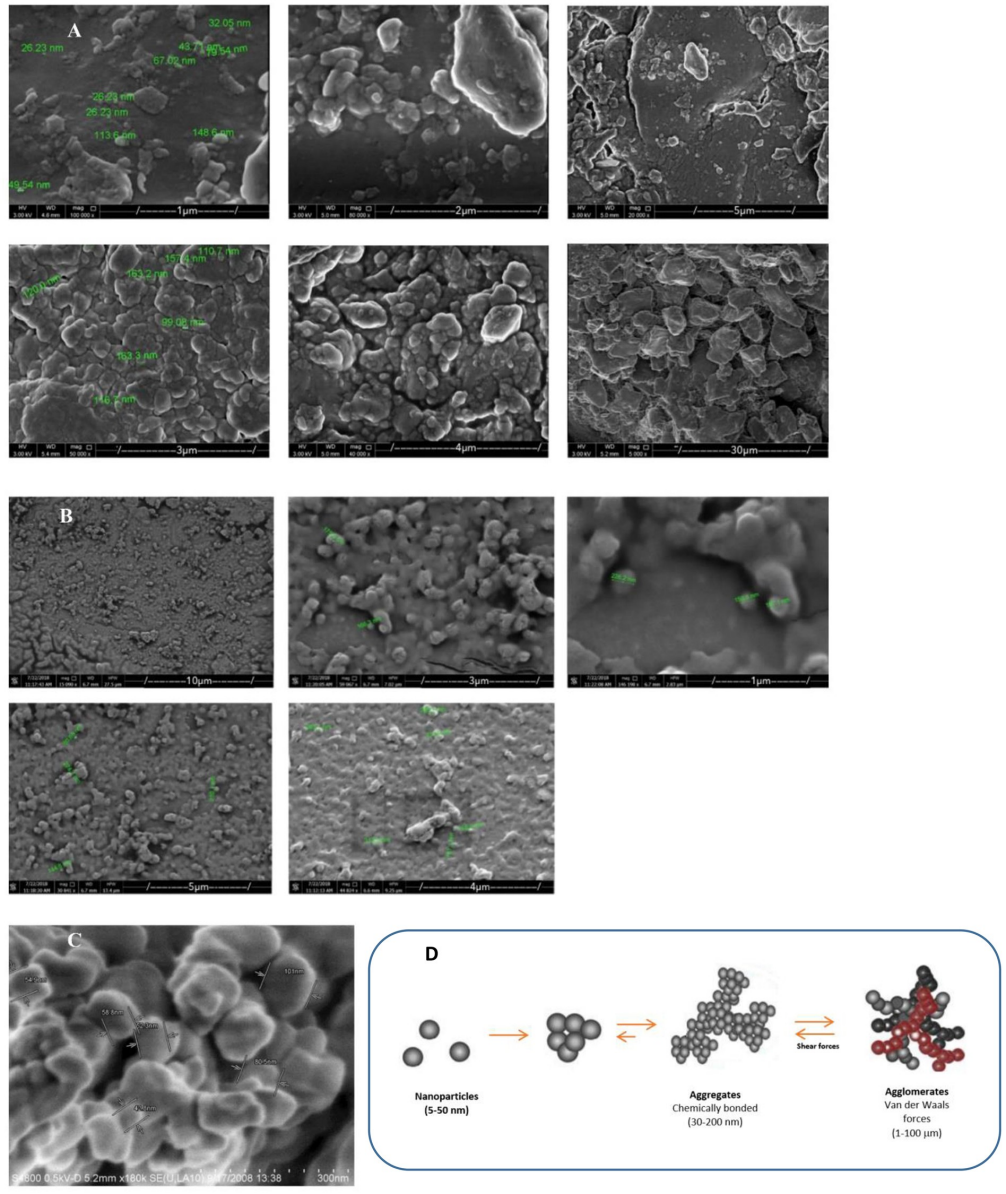
**A**. SEM images of the prepared DP nanoparticles, **B**. SEM images of sample particles suspended in ethanol and left to dry on a glass slide. Agglomeration was visible, masking smaller particles, **C**. Severe degree of agglomeration of nanoparticles that provides the representation of particle morphology and size very difficult [47], and D. Particle aggregation/agglomeration [43].

Fig 6 shows the SEM images of the prepared DP nanoparticle. Agglomeration was visible, masking smaller particles. According to [22], the particle agglomeration could be due to the presence of *hydrogen bonds between the particles of the natural fillers*. As the smaller particles have large surface areas, the possibility of aggregation/agglomeration will be increased and would enhance the possibility to interact with the other particles [43]. Furthermore [44], suggested that fiber agglomeration can be reduced by using hydrophobation treatments that weaken the hydrogen bonds between the natural fibers. The agglomeration state and the stability state of the nanoparticles can be determined by the sum of the repulsive and attractive forces between individual nanoparticles. The attractive forces between the nanoparticles are a result of van der Waal forces. Cellulose nanoparticles usually form an interaction of electric double layers that coat each particle, which is called an electrostatic repulsive force [45]. Two critical characteristics of the electric double layer are the zeta potential and the thickness of the electrical double coating [46]. Fig 3C shows an agglomeration where the identification of the size and the shape of particles are almost not possible [47].

A good nanoparticle dispersion is considered as one of the main challenges in producing nanoparticles due to the restriction of interfacial area. The nanoparticle aggregation/agglomeration may reduce the final mechanical properties and the qualitatively changes the behavior of the final product. This could be achieved by reducing the attractive forces between the nanoparticles [48]. Coating the particles with a high molecular weight polymer would enhance the surface chemistry of the particles, stabilize the particles against aggregation/agglomeration, reduce the inter-particle dipole-dipole forces, and increase the hydrodynamic size of the particles [47].

#### 3.1.2. Thermophysical properties

**Fig 7:**
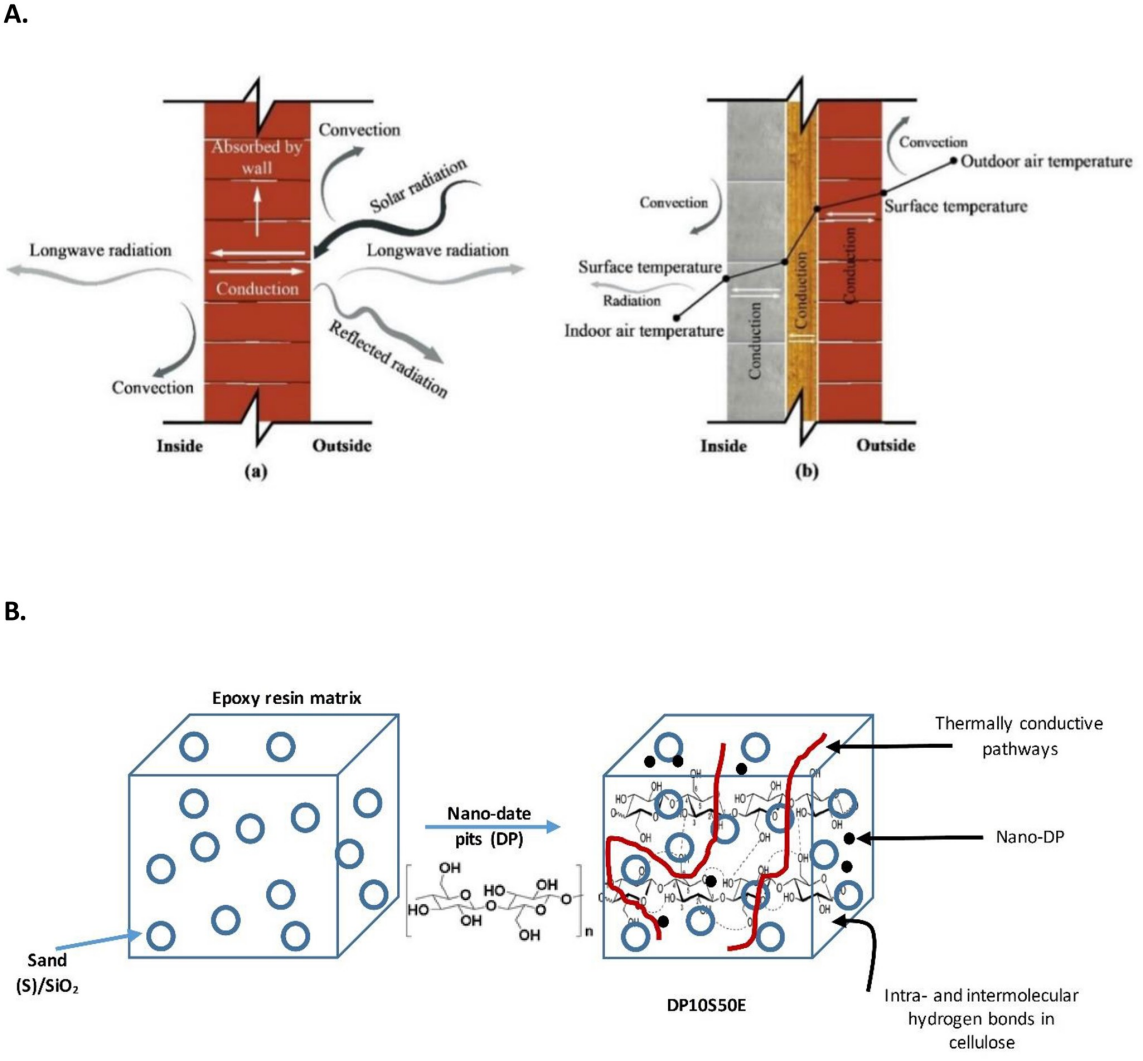
A. Heat transfer process across the solid wall and composite wall [49], and B. Schematics of the three-dimensional thermal conductive network formation for Sand (S)/SiO_2_ Particles and Nano-date pits (DP).

The thermal conductivity (λ), density (ρ), and thermal diffusivity (α) are the three key thermophysical properties required for the thermal behavior analysis and understanding for the heat exchange rate (conduction, convection, and radiation) and direction through building. Fig 7A illustrates the heat transfer process across the solid wall and composite wall.

As presented in Table 2, the S-to-DP ratio has a positive effect on the thermal conductivity (**λ)**. The ratio of DP30S and DP40S were having the same thermal conductivity. The samples formed an agglomerate with high porosity, and this was due to the poor filling of the pores and voids that carry air. Thermal diffusivity and penetration are directly proportional [5], the lower the thermal diffusivity, the lower the penetration. Low thermal diffusivity values are good for minimizing heat and thermal conduction. Nguyen et al. (2018) [50], used the bamboo particleboard powder as a novel bio-insulator and found that the same thermal conductivity trend was also observed. The bamboo particles having a diameter of 0.1–0.2 mm were crushed from bamboo fibers, having a value of 0.101 W/mK. However [51], examined the effect of placing the formulation polyester—filler composite as insulation material using waste rubber particles as filler in ratio 0–40% volume, results showed a low value of thermal conductivity 0.144–0.113 W/mK. A low thermal conductivity (**λ)** is desired for energy efficiency in buildings as the thermal insulation strongly affects energy performance. The heat storage plays a key role if the thermal conductivity of the material is 3.0 W/mK or higher. However, if the thermal conductivity of the material is lower than 0.3 W/mK, then the heat storage role vanishes [52]. For instance, the average thermal conductivity of the DP0S50E was 0.2626 W/mK.

**Table 2:** Thermophysical characterizations of the samples.

Material	Average Thermal Conductivity, λ (W/mk)	Average Thermal Diffusivity, α (m^2^/s)	Standard Deviation, σ	Density, *ρ* (kg⁄m^3^)
DP0S	0.0960	0.7050	0.0003	0.8410
DP10S	0.0924	0.1980	0.0001	0.9385
DP20S	0.0831	0.4029	0.0006	0.9438
DP30S	0.0796	0.2520	0.0003	0.8943
DP40S	0.0786	0.3235	0.0002	0.8943
DP0S50E	0.2626	0.1636	0.0001	0.9500

Fig 7 shows the schematics of the three-dimensional thermal conductive network formation for sand (S)/SiO_2_ particles and nano-date pits (DP). The very stable β (1→4)-link is strengthened by the followings: (i) intrachain H-bonds between the C3 (-OH) group and the adjacent in-ring oxygen, and (ii) between the C2 (-OH) group and the hydroxyl methyl group oxygen on C6 [20].

Furthermore, the thermophysical properties of DP0S50E, DP10S50E, DP20S50E, DP30S50E, and DP40S50E revealed that the average thermal conductivity for each composition is as shown in Table 2. Each reading was repeated three times to eliminate the reading’s error. As illustrated in Table 2, the S-to-DP ratio is no longer effective at insulating. The density of polymers was found using Eq 4. The obtained densities of the prepared polymers; DP0S50E, DP10S50E, DP20S50E, and DP30S50E were 0.997 kg⁄m^3^, 1.026 kg⁄m^3^, 0.99 kg⁄m^3^, and 1.000 kg⁄m^3^, respectively.

Additionally, the thermal diffusivity (α, m^2^/s) of a sample relates to the unsteady state of heat transfer and indicates how fast the material temperature reaches thermal equilibrium with the surrounding [49]. α with a high value shows faster heat spread through the sample. For instance, DP0S50E has the lowest thermal diffusivity, which is 0.1636 m^2^/s.

$$\rho =\frac{\lambda }{\alpha ∙C_{p}}$$ (5)

Where ρ is the density (kg⁄m^3^), λ is the thermal conductivity (W⁄mK), α is the thermal diffusivity (m^2^⁄s), and C_p_ is the specific heat capacity ($\frac{J}{kg.K}$).

Fig 7 shows that the improvement in through-thickness thermal conductivity is highly related to the distribution, organization, and connectivity of DP within the epoxy matrix. As for the effect of the nanosized-DP, the composites with cellulose networks showed lower thermal conductivity [53]. The changes in thermal conductivity with DP content were consistent with percolation theory when using the DP/cellulose as a filler. The thermal conductivity values with the DP exhibited lower thermal conductivities than that of epoxy resin fabricated with sand only. This showed that thermally conductive pathways had formed in the formed composites. Table 3 shows the comparison among thermal conductivity enhancement in some polypropylene (PP)—based materials due to the effect of different introduced fillers into the matrix.

**Table 3:** Thermal conductivity of various polypropylene (PP)–based materials with different fillers introduced into the matrix.

Materials	Thermal conductivity of Polypropylene (W/mK)	Filler content	Thermal conductivity of Polypropylene -composites (W/mK)	Reference
PP/Cu	0.25	35%	2.20	[54]
PP/carbon nanofiber	0.125	8%vol	0.181	[55]
PP/MWNT	0.09	5 vol%	0.20	[33]
PP/boron nitride	0.224	4%vol	0.629	[56]
PP/boron nitride	0.22	12%vol	0.47	[57]
PP/maleic anhydride grafted	0.22	25%	0.58	[57]
Polypropylene/expanded graphite	-	20%vol	1.1	[58]
Polypropylene/multi-wall carbon nanotubes	-	20%vol	0.75	[58]
Polypropylene/expanded graphite/multi-wall carbon nanotubes	-	20%vol	1.5	[58]
Polypropylene/carbon nanotubes	-	4%vol	0.35	[59]
Polypropylene/graphene nanoplatelets	-	16.7%vol	0.85	[60]

#### 3.1.3 UV aging test

**Fig 8:**
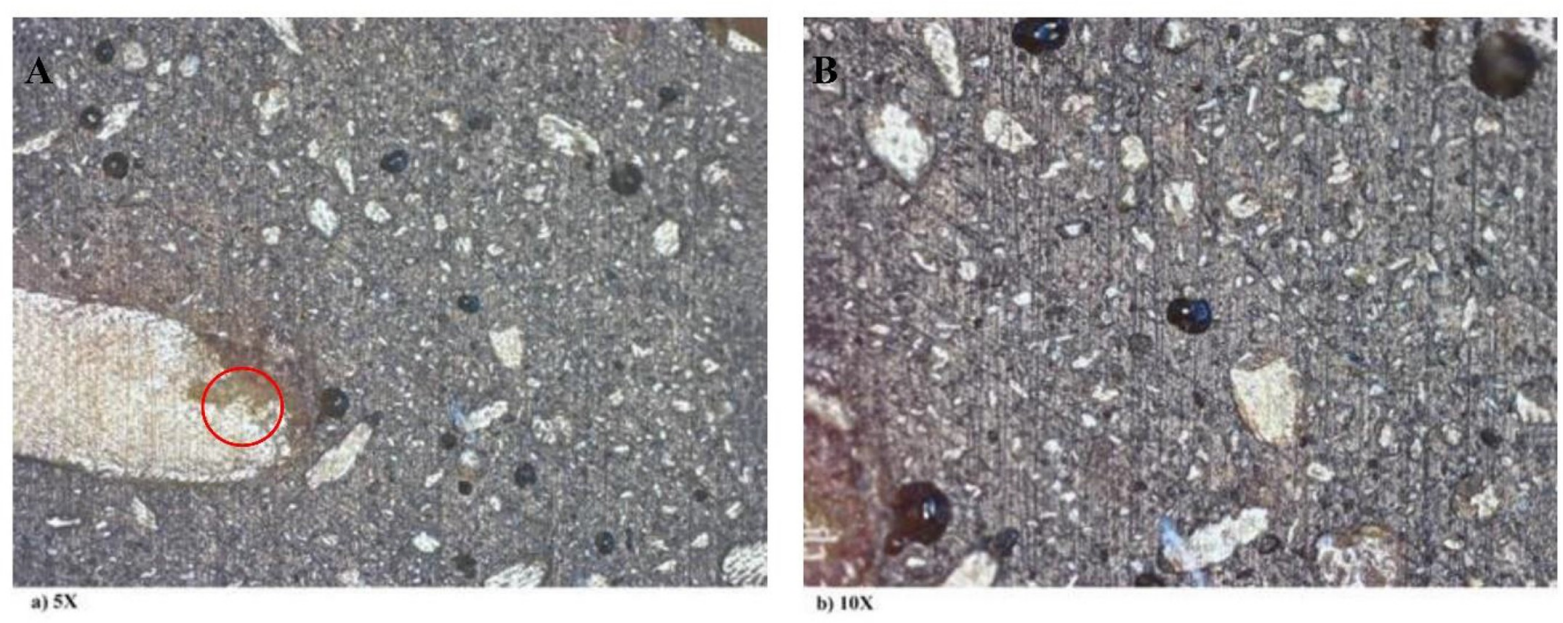
Microscopic images marking the air voids of DP0S50E with a) 5X magnification and b) 10X magnification.

**Fig 9:**
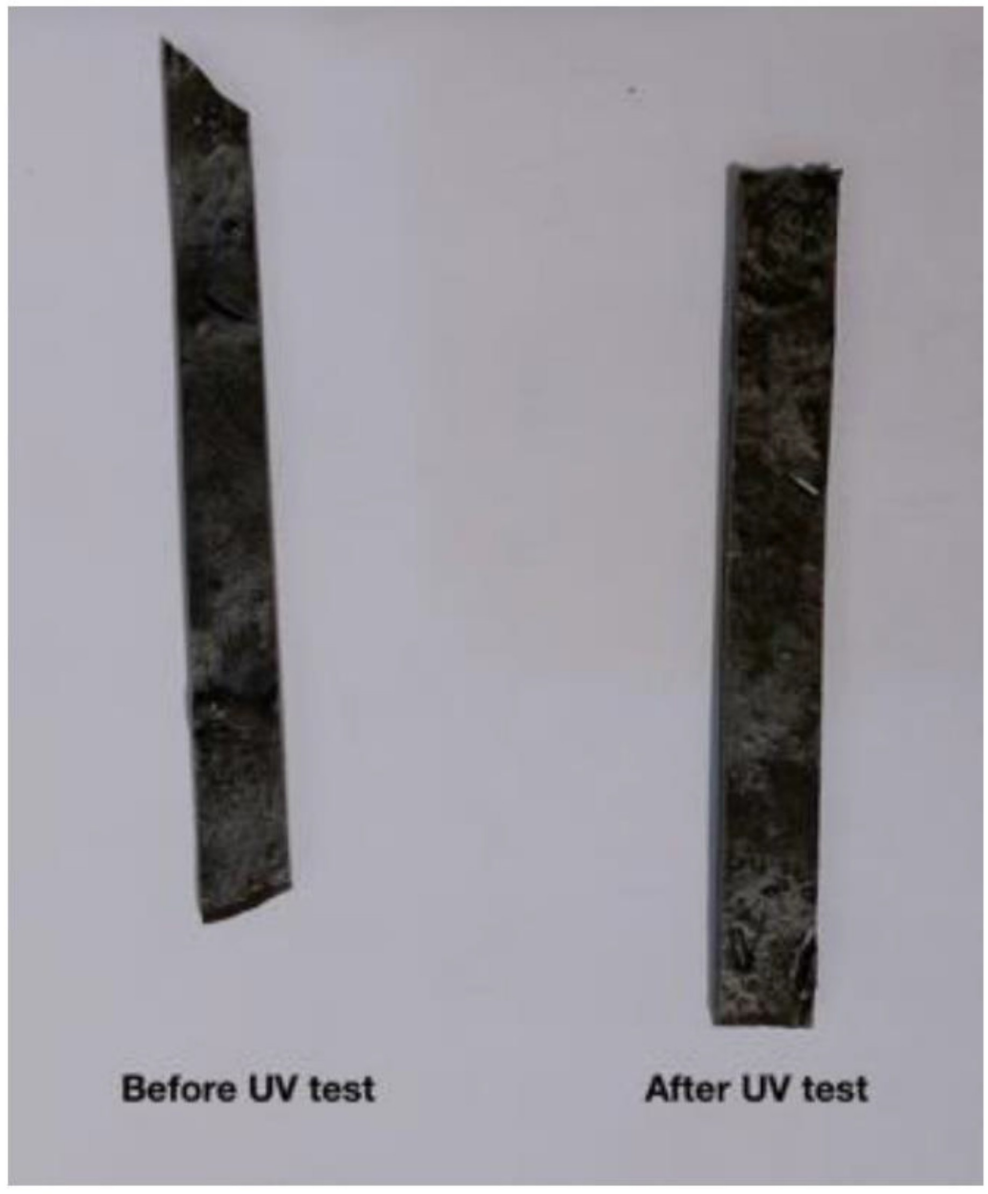
Changing in color pigmentation before and after UV aging test for DP0S50E.

Since DP0S50E is a polymer of epoxy and nanoparticles of DP with a ratio of 1:1 and characterized by its lightweight, low density, low thermal conductivity, as shown in Table 2. Moreover, DP0S50E has low thermal conductivity which indicates that it is a good insulator as its reduced thermal diffusivity increases its strength, allowing slower heat penetration. Therefore, DP0S50E was chosen as a sample to perform the UV aging test. Fig 8 shows the images of DP0S50E before exposure to the UV aging test with two different magnifications: 5X and 10X. The images illustrated the DP particles with few voids due to the traditional method of polymer composite preparation. The microscopic photos showed a semi-homogenous mixture of DP0S50E with no observed changes. The discoloration (i.e. yellowing) observed with exposure time is due to a reduction in spectral reflectance over the spectral range. The DP shields the material from UV light aging. Visual inspection can provide information as to the state of a material’s surface (i.e. color changes, crazing, and resin loss). UV irritation destroys the cellulose according to findings. Fig 9 illustrates the color change in pigmentation of for DP0S50E before and after applying the UV aging test.

**Fig 10:**
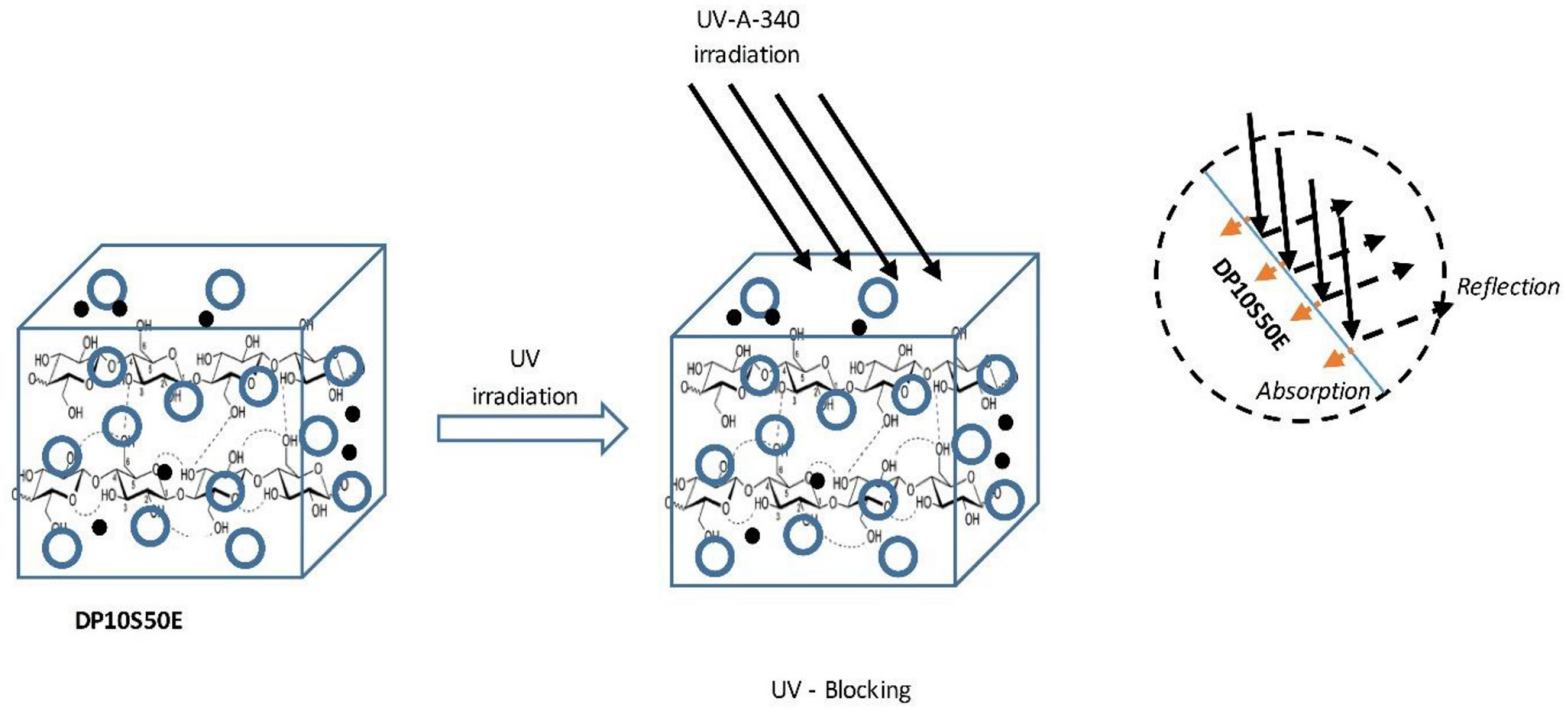
Possible photostabilization mechanisms of DP10S50E.

**Fig 11:**
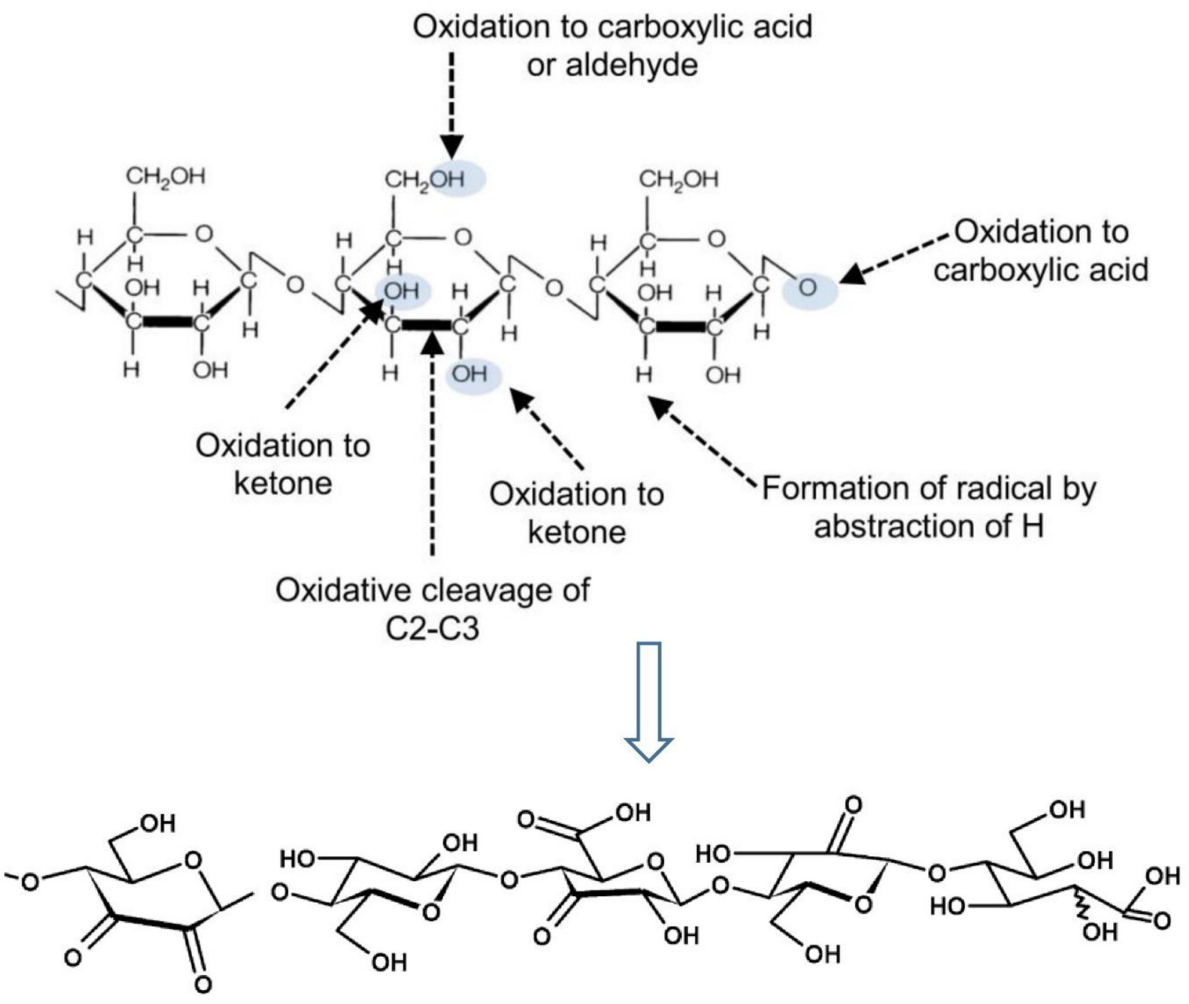
The positions in the cellulose chain possible to participate in oxidation reactions [61,62].

Fig 10 illustrates the possible photostabilization of DP10S50E. A part of UV irradiation could be reflected by the DP10S50E surface as a physical shielding effect. However, some of the transmitted irradiation was absorbed by the DP10S50E. As a result, the formed free radical scavengers will then oxidize the DP/cellulose. The chromophore groups in the oxidized products from the cellulose led to severe color changes [61]. Moreover, Fig 10 depicts the reinforced composites containing the UV-absorbing DP10S50E. It shows the utilization of cellulose-based materials as a filler and UV blocking. The incorporation of photoactive cellulose-based materials could improve the transparency and UV-absorption properties of the materials. Fig 11 shows the cellulose possible positions to contribute to oxidation reactions [61].

#### 3.1.4. Fourier transform infrared spectroscopy (FTIR)

The FTIR of the samples (DP0S50E, DP10S50E, DP20S50E, and DP30S50E) were investigated. The broad peak at 3000–3600 cm^-1^ was attributed to–OH functional groups. The appearance of the–NCO peak at 2240 cm^-1^, the C = O peak at 1700 cm^-1^ and the reduction of the–OH peak were revealing of the modification of –OH groups onto the surface of the nano-DP [26]. The wavelength 827 cm^-1^ indicates for C–H 1,4-disubstituted or 1,2,3,4-tetrasubstituted, the peak at 915 cm^-1^ is due to the presence of alkene C = C monosubstituted, the observed band at 1250–1050 cm^-1^ is assigned to C-O-C stretch group, the band ranging from 1600–1400 cm^-1^ may be due to the NO_2_ stretch or C = C aromatic. The 1607 and 1744 cm^-1^ is due to carboxylic acid C = O stretch and the broad and strong band were situated at 2900–2800 cm^-1^, which is attributed to the C-H aldehyde. Wide bands that are visible at 1100–1250 cm^-1^ are attributed to the quartz in the sand [63].

The FTIR peak identifications were supported by a study carried out for heavy metals and dye adsorption techniques [64]. It was shown that the 1744 cm^-1^ indicates the unconjugated C-O in xylan (hemicellulose), 1449 cm^-1^ for C–H found in lignin and carbohydrates, 1246 cm^-1^ for syringyl ring, and C–O in lignin and xylan. The 1056 cm^-1^ was assigned for C–O–C vibration in cellulose and hemicellulose, 1058 cm^-1^ attribute to C–O stretch in cellulose and hemicellulose, and 869 cm^-1^ due to C–H distortion in cellulose. Similarly, another study investigated dunes sand in Ouargla, Algeria. The study also demonstrated the FTIR peak identification [63]. The peaks that match are found in the following frequencies, the wideband between 1100–1250 cm^-1^ due to the quartz in the dunes sand and the peak at 1607 cm^-1^ due to gypsum. As well as some organic compounds (C-H aldehyde) found in 2923–2852 cm^-1^.

#### 3.1.5. Thermogravimetric analysis (TGA)

**Fig 12:**
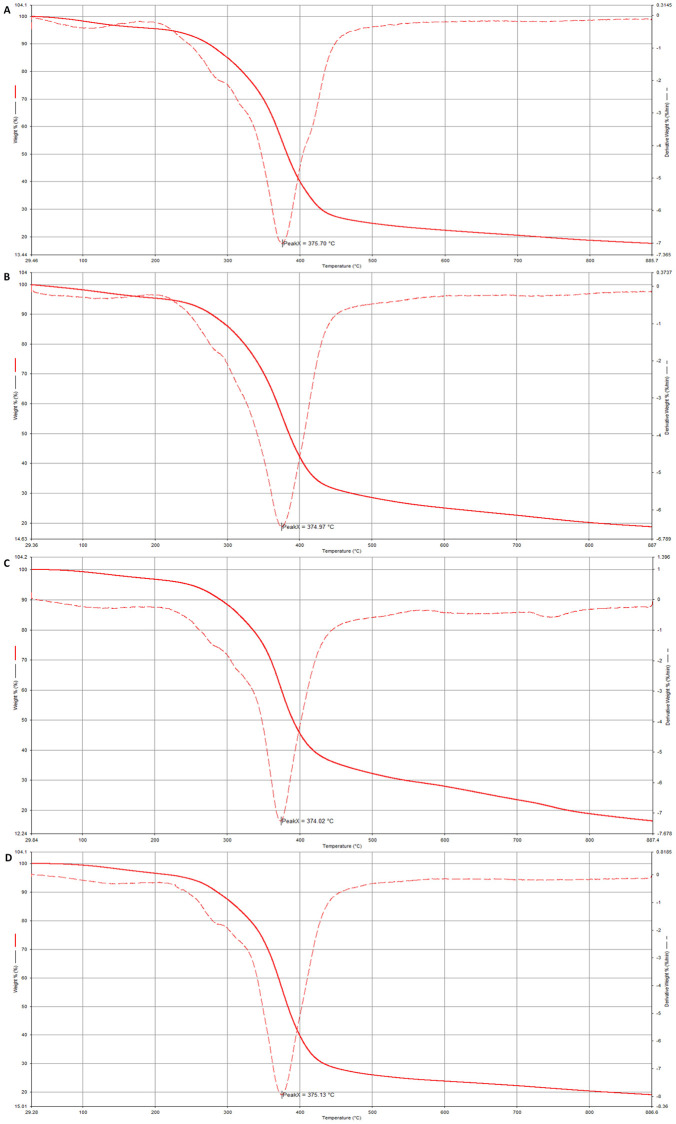
Average TGA curves and their first derivative curves for a) DP10S50E, b) DP20S50E, c) DP30S50E, and d) DP40S50S.

The thermograms of the samples exhibited the weight loss of the specimens. It was shown that the TGA curves and their derivatives could be used to determine the exact temperature ranges of weight loss of specimens. The TGA peaks represented 50% of the weight loss of each sample. A study was performed to investigate thermal degradation for an epoxy polymer using nano bio-filler to enhance fire protective performance [65]. It was noticed that the distinct peaks were at 375.70°C, 374.96°C, 374.02°C, and 375.13°C for DP10S50E, DP20S50E, DP30S50E, and DP40S50E, respectively as shown in Fig 12. The peak was marked at 370.47°C. The distinct step-wise dehydroxylation gives a clear indication of the neighboring group effect on the dehydroxylation steps. At a temperature range of 360–400°C, the endothermic degradation of the condensed hemiketal groups will occur. The first derivative TGA did not show any impression of this stepwise degradation. To further characterize the samples, the extrapolated onset temperature (To), which signifies the temperature at which the weight loss starts, was calculated. The extrapolated onset Temperature was 345°C.

**Fig 13:**
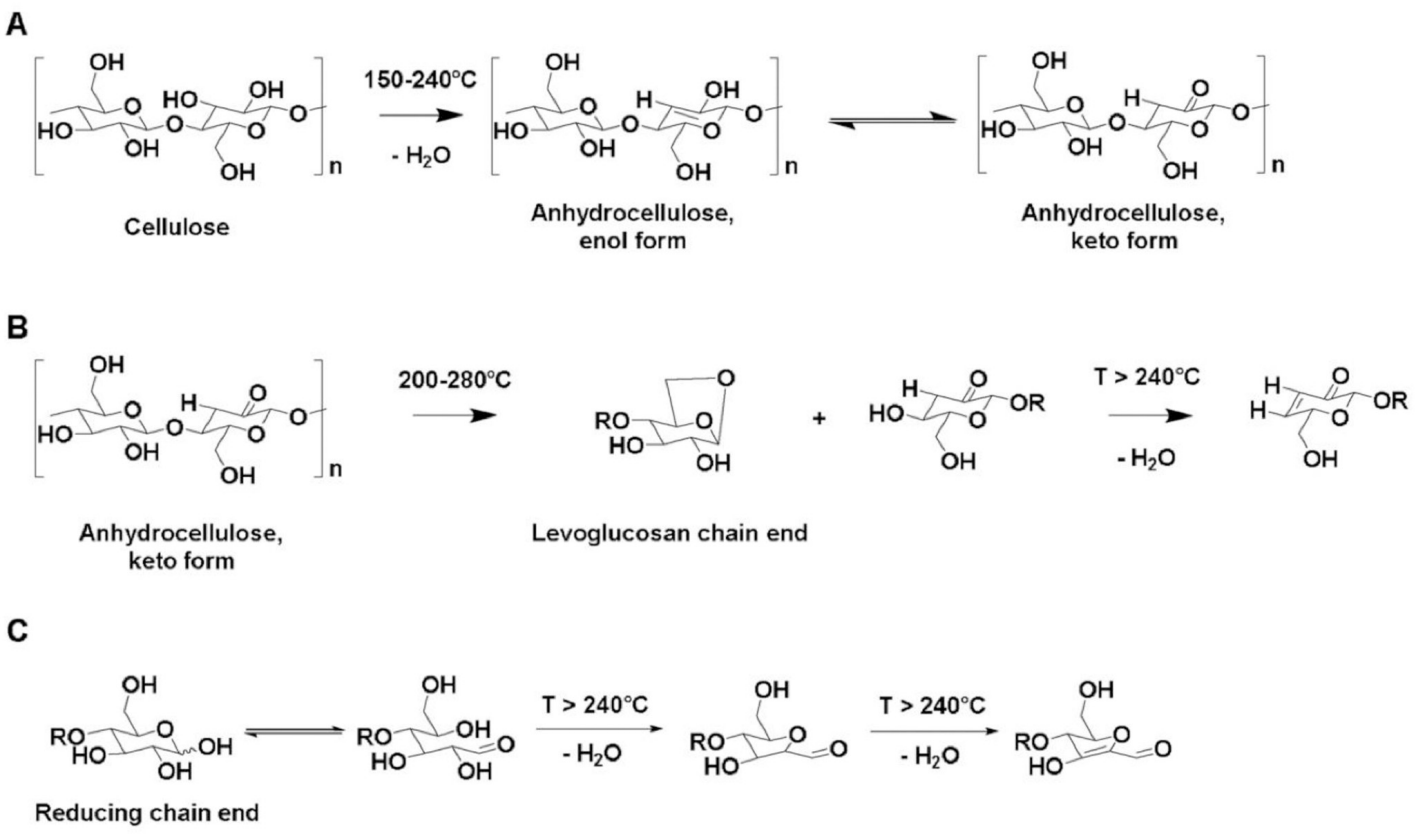
A. Thermal dehydration of amorphous cellulose from 150 ◦C to 240°C, B. Thermal dehydration of amorphous cellulose from 200 ◦C to 280°C, and C. Thermal dehydration of reducing end of celluloses, R = cellulose residue [66].

Our results were similar to the results obtained by [65], who studied the thermal degradation of epoxy polymer using a novel eggshell as a nano bio-filler. The polymers lost 50% of their original weight recorded for temperatures below 400°C. Therefore, we can conclude that date pits used as nanomaterials might be applied for better fire protective performance. Fig 13 shows the ways of water release from cellulose at different temperatures [66]. Significant amounts of water will be released from the amorphous cellulose fraction at the thermal degradation of cellulose by a random cleavage of the cellulose chain.

### 3.2. Building energy simulation

**Fig 14:**
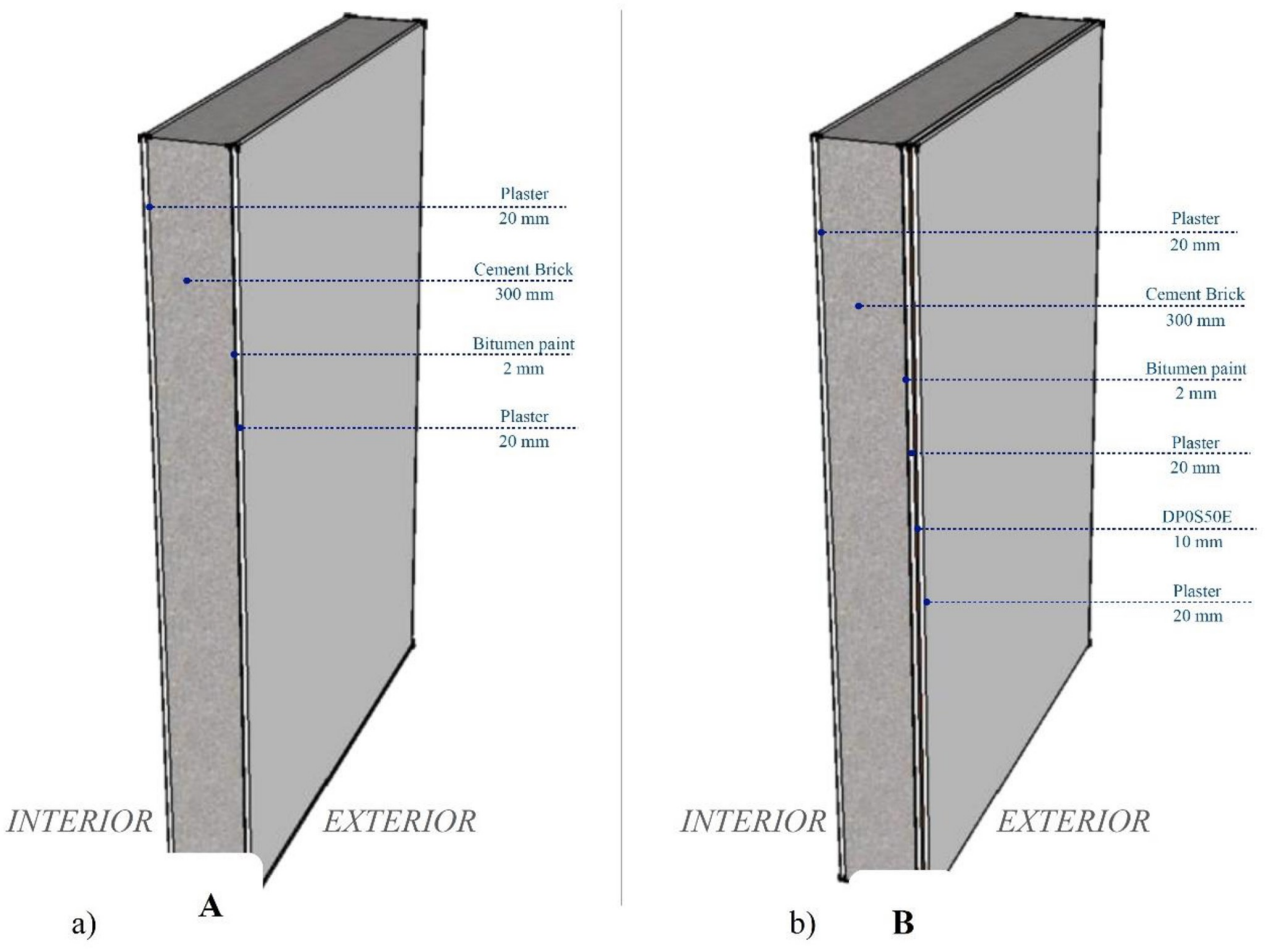
3-D sections illustrate **A**. the layers in typical Qatari houses and **B**. how to locate the proposed material in existing houses.

The energy usage of buildings varies greatly from one building to another [67]. Operating a good building simulation focuses on weather data and building masses, while other parameters that control the energy usage in buildings are building operation, maintenance, occupant behavior, and the activities inside the building [68]. The simulation was done using Autodesk Revit 2017 with a small house design with an area of 570 m^2^ and an input location of 25.28152°N, 51.39164°E. Three simulation models were running: a house with typical wall layers TH1 and a house with added DP0S50E as a layer in exterior walls, coded as PHDP. The exterior wall area for all models was 506 m^2^. The 3-D section of the exterior walls in typical Qatari houses is shown in Fig 14, with a comparison of layers in exterior walls of existing houses using the proposed materials.

Based on previous and recent literature building energy simulation is broadly carried in research [69–72]. Many software programs were used in the simulation of the energy performance of different building types to analyze the performance of different materials programmatically. Table 4 summarizes and compares all the relevant information analyzed using building energy simulation. All the evaluated models estimate energy consumption, where the highest performance in saving energy was demonstrated by [69]. They used thermally enhanced sustainable hybrid brick and as a result, the usage of energy decreased by 9.40%. Although the previously mentioned studies conducted their research on new buildings, our proposed model in this thesis relied on existing buildings aiming to save time and cost. Previous research studied the enrichment of having new modified insulation material using a nano vacuum insulation panel, where the panels should be installed between two 100 mm bricks on the external walls, although the usage of energy decreased by only 0.57% [70]. These results strengthen the ability to use DP0S50E as insulation material for the coating of existing buildings.

**Table 4:** Summarizes and compares all the relevant information analyzed using building energy simulation.

Research Building Type	Area (m^2^)	Location	Software	Aim	Type of modification	Energy-saving potential (%)	Applicable in existing building?	Reference
Residential House	570	Qatar	Autodesk Revit	Cooling energy and carbon emission	Nanoparticles of date pits insulation on external walls only	6.49	yes	Present study
Office Building	200	Saudi Arabia	Autodesk Revit and Autodesk Ecotect	Cooling energy and total energy consumption	Nano-vacuum insulation panels on external walls only	0.57	no	[70]
Residential House	234	Malaysia	Ansys	Energy consumption, electricity consumption and carbon emission	Thermally enhanced sustainable hybrid brick	9.40	no	[69]
Residential House	400	Malaysia	Autodesk Revit and Autodesk Ecotect	Operational energy consumption	Double brick cavity plaster	6.02	no	[72]
20 Story Housing	70,824	Korea	IES VE	Operational energy and life cycle cost	Glass fiber reinforced concrete	0.43	no	[71]
20 Story Housing	70,824	Korea	IES VE	Operational energy and life cycle cost	Cellulose fiber reinforced concrete	0.55	no	[71]

#### 3.2.1. Energy usage simulation of TH1 model

**Fig 15:**
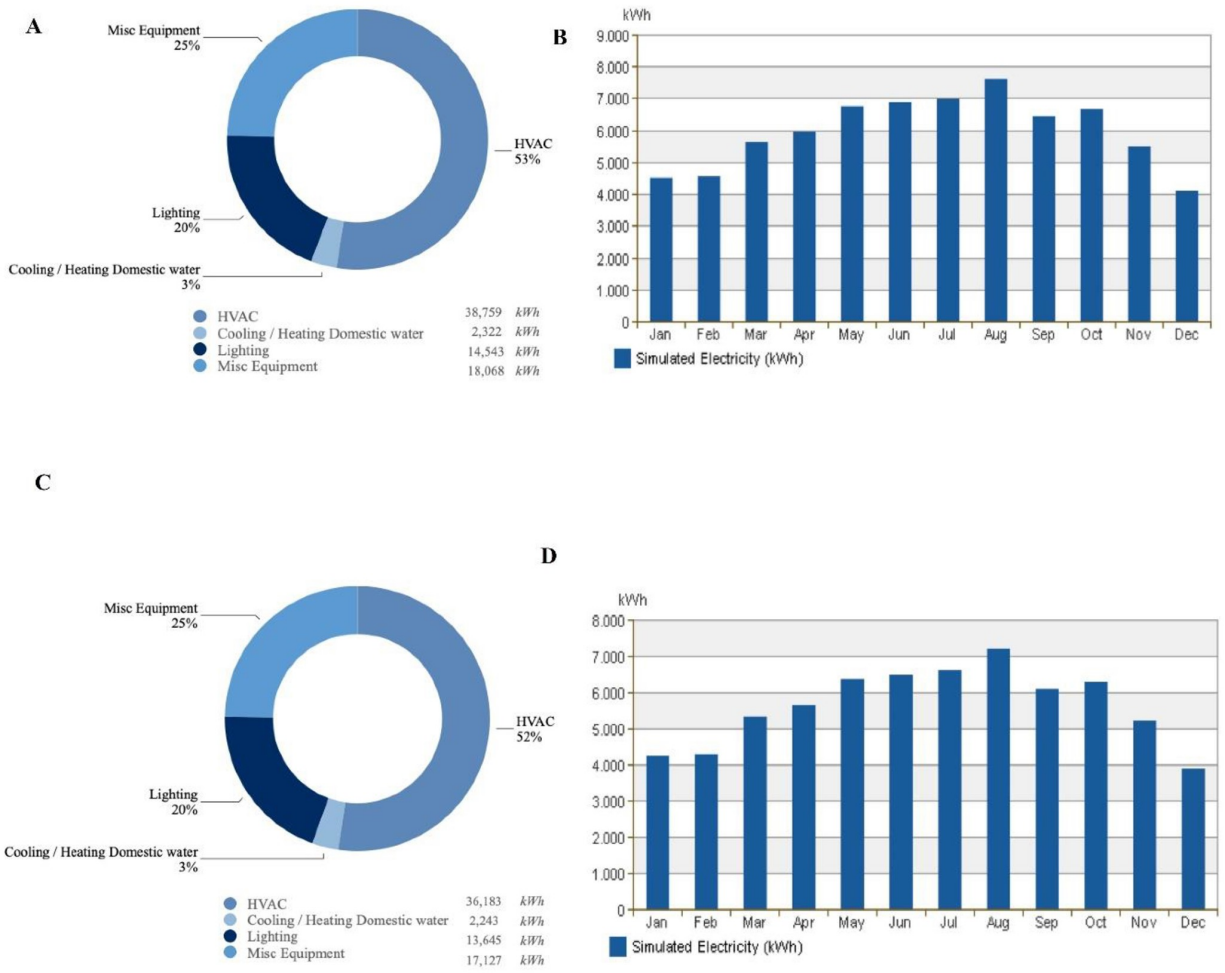
**A**. The breakdown of annual energy usage for TH1, **B**. The monthly electricity consumption of TH1, **C**. The breakdown of annual energy usage for PHDP, **D**. The monthly electricity consumption by PHDP.

Simulating TH1 shows that the regular Qatari house with an area of 570 m^2^ tends to use 73,692 kWh per year of energy. A 53% of this energy is used to cool down the house to reach a comfortable indoor temperature, as presented in Fig 15A (a breakdown of energy use building as given by an Autodesk Revit 2017 simulation). Fig 15B shows the monthly electricity consumption by TH1; showing that May through August has the highest energy usage.

#### 3.2.2. Energy usage simulation of PHDP model

The simulated model of PHDP shows that the annual energy consumption of the same 570 m^2^ house using DP0S50E, as a coating material on the external walls, is 69,198 kWh, which is 4,494 kWh less than TH1, meaning it is 6.49% more energy-efficient than TH1. Saving 4,494 kWh of energy means reducing the CO_2_ emitted, by a house with an area of 570 m^2^ by 1.8 tons annually (calculated based on the Emission Factors in 400 gCO_2_/kWh). Fig 15C shows that 52% of energy consumption is a result of using the HVAC system. The monthly energy usage by PHDP is shown in Fig 15D, wherein the month of August, the highest energy usage was used, to cool down the building to a comfortable indoor temperature.

Given that the price of electricity for household buildings in Qatar is $0.032/kWh [73], this is equivalent to annual energy cost savings of $144.

## 4. Conclusion

Nanoparticles that can be used as coatings for architectural purposes are known to provide suitable adhesion and protection against fire and corrosion. Moreover, they can be used to incorporate thermal insulations in buildings. Therefore, date pits usage as a nanomaterial proved that they play a pivotal role in the building industry. DP0S50E was chosen as a model example. The microscopic photos showed a semi-homogenous mixture of DP0S50E with no observed changes. The discoloration (i.e. yellowing) observed with exposure time is due to a reduction in spectral reflectance over the spectral range. The average thermal conductivity of the DP0S50E was 0.2626 W/mK, and it has the lowest thermal diffusivity, which is 0.1636 m^2^/s. The DP0S50E has a low thermal expansion characteristic, which will give it more durability for its usage.

It was proposed that polymer DP0S50E can be used as a coating material for existing buildings with a thickness of 10 mm. The results prove that polymer DP0S50E has proven to be efficient regarding energy usage by 6% and eliminating the emission of CO_2_ by 6%. Although DP0S50E was used in an existing building, rather than in the construction of a new building, thus it allows the polymer to reduce the cost of energy generation and its impact on the environment. Given that the price of electricity for household buildings in Qatar is $0.032/kWh [73], this is equivalent to annual energy cost savings of $144.

Moreover, FTIR, SEM, and TEM were used to characterize the materials used, where the nanoparticle material was chosen as the most suitable thermal insulator. Those polymeric insulators are now a trend in the replacement of coating insulations. The use of this promising technology offers many advantages to the construction sector; nevertheless, in a polluted environment with high humidity levels, (i.e. Qatar, as a case study), nanoparticles affect the coating surface of the insulation. However, with the limited amount of filler that can be added for the processability of the epoxy, it becomes more difficult and expensive in cost. Some examinations should be followed in the future, such as study the flammability, how to extract the oil from the nanoparticle of the date pits, and the mechanical properties of the material itself. In conclusion, the future of using date pits in construction is bright and promising due to their special characteristics.
